# Development of a Combat-Relevant Murine Model of Wound Mucormycosis: A Platform for the Pre-Clinical Investigation of Novel Therapeutics for Wound-Invasive Fungal Diseases

**DOI:** 10.3390/jof10050364

**Published:** 2024-05-20

**Authors:** Rex J. R. Samdavid Thanapaul, Yonas A. Alamneh, Daniel K. Finnegan, Vlado Antonic, Rania Abu-Taleb, Christine Czintos, Dylan Boone, Wanwen Su, Venkatasivasai S. Sajja, Derese Getnet, Ashleigh Roberds, Thomas J. Walsh, Alexander G. Bobrov

**Affiliations:** 1 Wound Infections Department, Bacterial Diseases Branch, Center for Infectious Diseases Research, Walter Reed Army Institute of Research, Silver Spring, MD 20910, USA; 2 NRC Research Associateship Programs, National Academies of Sciences, Engineering, and Medicine, Washington, DC 20001, USA; 3 Veterinary Services Program, Pathology Department, Walter Reed Army Institute of Research, Silver Spring, MD 20910, USA; 4 Blast Induced Neurotrauma Branch, Center for Military Psychiatry and Neuroscience, Walter Reed Army Institute of Research, Silver Spring, MD 20910, USA; 5 Departments of Medicine and Microbiology & Immunology, University of Maryland School of Medicine, Baltimore, MD 21201, USA; 6 Center for Innovative Therapeutics and Diagnostics, Richmond, VA 23220, USA

**Keywords:** combat wound-invasive fungal disease, mucormycosis, *Rhizopus arrhizus*, *Lichtheimia corymbifera*, liposomal amphotericin B, mice

## Abstract

Wound-invasive fungal diseases (WIFDs), especially mucormycosis, have emerged as life-threatening infections during recent military combat operations. Many combat-relevant fungal pathogens are refractory to current antifungal therapy. Therefore, animal models of WIFDs are urgently needed to investigate new therapeutic solutions. Our study establishes combat-relevant murine models of wound mucormycosis using *Rhizopus arrhizus* and *Lichtheimia corymbifera*, two *Mucorales* species that cause wound mucormycosis worldwide. These models recapitulate the characteristics of combat-related wounds from explosions, including blast overpressure exposure, full-thickness skin injury, fascial damage, and muscle crush. The independent inoculation of both pathogens caused sustained infections and enlarged wounds. Histopathological analysis confirmed the presence of necrosis and fungal hyphae in the wound bed and adjacent muscle tissue. Semi-quantification of fungal burden by colony-forming units corroborated the infection. Treatment with liposomal amphotericin B, 30 mg/kg, effectively controlled *R. arrhizus* growth and significantly reduced residual fungal burden in infected wounds (*p* < 0.001). This study establishes the first combat-relevant murine model of wound mucormycosis, paving the way for developing and evaluating novel antifungal therapies against combat-associated WIFDs.

## 1. Introduction

Wound infections remain a critical threat to military personnel on the battlefield where conventional explosives and improvised explosive devices continue to be a prominent cause of injuries, often resulting in extensive tissue damage, embedded foreign objects, and severe blood loss [1,2,3,4,5]. These insults cause immune dysregulation and create an environment conducive to infection [6,7]. Injuries from blasts are classified into five categories: (i) primary blast injuries (PBIs), which result from overpressure/shock wave, (ii) secondary blast injuries (SBIs), which are resultant of shrapnel and/or debris dislodged or energized from the explosive event, (iii) dislodging of the subject and hitting an object or the ground, (iv) quaternary blast injuries, resultant of burns and/or toxic gases, and (v) quinary blast injuries, which are not associated with the first four categories and are resultant of chemical wounds, radiation, etc. [8].

Blast-penetrating trauma, which is a combination of PBI and SBI, commonly leads to post-traumatic wound-invasive fungal diseases (WIFDs), which continue to exhibit alarmingly high mortality rates, reaching up to 8% during U.S. military operations in Afghanistan from 2001 to 2021 [9,10,11,12]. Early reports of a WIFD outbreak amongst wounded members of the Israel Defense Forces during operations in Gaza suggest that the WIFD threat remains a global military concern and that mortality rates have not improved (at least two out of ten infected soldiers were reported to succumb to WIFDs) (published in The Jerusalem Post). *Mucorales* species (spp.) are the primary causative agents of WIFDs among military personnel. Mucormycosis often leads to angioinvasion, resulting in extensive tissue necrosis, non-healing wounds, and fatalities. Treating WIFDs, particularly invasive mucormycosis, in military personnel demands intensive care, frequent debridement, prolonged hospitalization, and, in severe cases, limb amputation [9,13,14,15,16]. The antifungal agents available for the treatment of WIFDs include liposomal amphotericin B (L-AmB) and triazoles such as posaconazole (POS), voriconazole (VOR), and isavuconazole (ISZ) [9,17,18]. Commonly, L-AmB has been favored to treat military mucormycosis, primarily due to the inherent VOR resistance of Mucormycetes and higher susceptibility of several *Mucorales* wound isolates to AmB compared to triazoles such as ISZ [9,19,20,21,22]. However, L-AmB has serious adverse side effects, including a high risk of nephrotoxicity. In addition, both ISZ and AmB have very limited clinical success, with 33–41% mortality rates despite treatment among people with confirmed invasive mucormycosis [17]. Thus, antifungal activity in the available treatments is suboptimal, and new therapeutic solutions to combat WIFDs are urgently needed to improve therapeutic outcomes.

Developing effective treatments for wound mucormycosis requires robust animal models that accurately mimic and are predictive of the disease in humans. Sporangiospores, the asexual reproductive propagules of *Mucorales* fungi, have been commonly used in pre-clinical animal models of pulmonary mucormycosis to evaluate potential therapies and elucidate pathogenesis. However, an extensive literature search failed to find specific publications on animal models of wound mucormycosis, and only limited studies have explored the pathogenesis and effects of different *Mucorales* spp. using pre-clinical animal models of cutaneous mucormycosis. Overall, only 10 reports were found on experimental cutaneous mucormycosis/zygomycosis in rodent and rabbit models. Sheldon and Bauer (1958, 1959, and 1960) and Paplanus and Sheldon (1963) employed subcutaneous and intradermal inoculation of *Rhizopus arrhizus* sporangiospore suspensions in rabbit and rat models under normal and diabetic conditions [23,24,25,26]. Other studies [19,20,21,22,27] investigated the effects of subcutaneous and intradermal inoculation of *Lichtheimia corymbifera*, *Rhizomucor pusillus*, *Mucor irregularis*, and *R. arrhizus* sporangiospores suspensions in mice. Lewis et al. (2013) evaluated the effects of subcutaneous injection of *R. arrhizus* sporangiospore suspensions in immunosuppressed mice [28]. Collectively, these studies aimed to replicate cutaneous mucormycosis using sporangiospores in controlled settings, but all failed to authentically replicate combat wound infections, which almost exclusively arise from blast wounds and uniquely involve direct environmental inoculation.

The absence of combat-relevant animal models for WIFDs presents a significant obstacle to the development of new interventions and adjunct therapies, and thus is a critical gap in military medicine. To initiate WIFDs mimicking the injuries seen in military personnel, we incorporated the following elements in our murine model. First, blast overpressure (BOP) exposure (primary blast) was used, since it has been demonstrated to cause organ/tissue damage and immune dysregulation [6,29,30]. Following BOP exposure, we induced a combined injury involving the skin, fascia, soft tissue, and muscle, mimicking primary and secondary blast injuries. Second, mice were infected with hyphae of *R. arrhizus* or *L. corymbifera*, both common causative agents of WIFDs worldwide [13,15,31,32]. Hyphae are a common element of fungal mycelial colonies inhabiting the soil and have been proposed to be the cause of WIFDs [15]. We recently demonstrated that hyphae have a 10- to 16-fold increase in virulence compared to sporangiospores in a *Galleria mellonella* model of traumatic mucormycosis [33]. Furthermore, we sought to validate our animal model and evaluate the efficacy of L-AmB against *R. arrhizus,* as it is typically utilized as an antifungal agent to mitigate the immediate threats posed by WIFDs.

## 2. Materials and Methods

### 2.1. Fungal Strain and Growth Conditions

*R. arrhizus* (ATCC 56536), isolated from the postmortem lung tissue of a male in the Netherlands with acute lymphoblastic leukemia, and *L. corymbifera* (ATCC 46771) isolated from soil in Afghanistan, were obtained from the American Type Culture Collection (ATCC), Manassas, VA, USA. Glycerol stocks of the strains were initiated for sporangiospore production on synthetic modified mucor agar (SMMA) or potato dextrose agar (PDA) plates and incubated at 37 °C for 7 days. Sporangiospores were isolated using a previously described method [33].

### 2.2. In Vitro Sporangiospore Germination Analysis

*R. arrhizus* or *L. corymbifera* sporangiospores (1.0 × 10^9^) were incubated in 5 mL of either yeast peptone dextrose broth medium (YPD) (Thermo Scientific, Waltham, MA, USA) or Roswell Park Memorial Institute (RPMI) medium supplemented with 10% or 20% fetal bovine serum (FBS) (Thermo Scientific, Waltham, MA, USA), or with a combination of 5 mM magnesium sulfate (Thermo Scientific, Waltham, MA, USA), 10 µM zinc chloride (Thermo Scientific, Waltham, MA, USA), and 10 µM ferrous sulfate under constant shaking at 150 rpm at 30 °C. At specified time intervals (3, 6, 9, 12, and 24 h), 20 μL of germling suspension was collected, examined under an Olympus BX53 light microscope, and counted using a hemocytometer (Nexcelom Bioscience LLC, Lawrence, MA, USA). The sporangiospore germination rate was calculated as the percentage of germinated sporangiospores relative to the total number of sporangiospores. The optimal conditions determined from this experiment were used for in vivo experiments.

### 2.3. Animals

All experiments were conducted in AAALAC-international accredited facilities under an approved Institutional Animal Care and Use Committee (IACUC) protocol, adhering to the Animal Welfare Act (AWA) and US Public Health Service (PHS) Policy on Humane Care and Use of Laboratory Animals. C57BL/6 mice were used, as these immunocompetent mice were reported to be acceptable hosts for sustained systemic or cutaneous *R. arrhizus* infections [20,34]. Adult male, 8-week-old C57BL/6 mice weighing 20 to 30 g were purchased from Charles River Laboratories, Wilmington, MA, USA, and housed at 21–23 °C with a 12 h light/dark cycle. All mice received sterile food and water ad libitum, and dry rodent chow was supplemented with DietGel^®^ Recovery (ClearH_2_O, Portland, ME, USA) during the 48 h following wounding. All mice were housed in groups of three in sanitized cages on sterile paper bedding and were provided with environmental enrichment, including in-cage plastic housing.

### 2.4. Inocula Preparations

Initially, for the lower inocula of *R. arrhizus* and *L. corymbifera* infections, we used RPMI with 10% serum, which was previously used for germination of *Rhizopus delemar* [35]. A low hyphal inoculum (~10^7^ CFU) was prepared by using ~10^9^ sporangiospores of either *R. arrhizus* or *L. corymbifera* inoculated into a 50 mL Falcon™ tube containing 5 mL of RPMI medium with 10% FBS and incubated with shaking at 30 °C, 150 rpm, for 24 h. Using this procedure, we were able to obtain more than 90% germination rate.

At the high sporangiospore concentration required for higher inocula preparations, the above-described RPMI method showed an 80% or less germination rate. To optimize sporangiospore germination, we tested various media conditions, including the YPD broth that was previously used for *R. arrhizus*, RPMI supplemented with increasing concentrations of FBS, and the addition of metal chelators. The addition of metals did not influence sporangiospore germination but stimulated fungal growth. Using an initial inoculum of 1 × 10^9^ CFU/mL *R. arrhizus* sporangiospores, the YPD medium provided a favorable sporangiospore germination rate of 90.29 ± 2.83% compared to 82.91 ± 2.47% for RPMI + 20% FBS (*p* = 0.015) (Figure S1A). Similarly, with an initial inoculum of 1 × 10^9^ *L. corymbifera* sporangiospores per mL, the YPD medium exhibited a higher sporangiospore germination rate of 98.59 ± 1.40% compared to 77.60 ± 8.51% for RPMI + 20% FBS (*p* = 0.006) (Figure S1B). One noticeable difference between the two media is that in RPMI, some sporangiospores germinate quickly and form very long hyphae, while in the YPD medium, swelling and germination progress more slowly but more uniformly). Perhaps long hyphae consume more nutrients, hindering the germination of the remaining fraction of non-germinated sporangiospores. Consequently, the optimized conditions using YPD medium were employed to generate high hyphal inocula (~10^8^ to 10^9^ CFU). Briefly, ~10^10^ to 10^11^ sporangiospores of *R. arrhizus* or *L. corymbifera* were inoculated into a 50 mL Falcon™ tube containing 5 mL of YPD broth medium and incubated with shaking at 30 °C, 150 rpm, for 9 h. Following incubation, the germling or hyphal suspension was centrifuged at 6000 rpm for 10 min, and the pellets were washed twice with 0.9% sterile saline. The pellet wet weight was recorded and diluted by adding 0.2 mL to 1 mL of 0.9% sterile saline. The inoculum was serially diluted, and the dilutions were plated on RPMI Agar plates and incubated at 30 °C overnight for colony-forming unit (CFU) validation.

### 2.5. Blast Overpressure Exposure, Surgery, Infection, and Treatment

For all animal experiments, mice were exposed to survivable blast overpressure (BOP) using a blast simulator located at the Walter Reed Army Institute of Research (WRAIR) described in detail elsewhere [29]. Briefly, after mice were anesthetized with 4% isoflurane for 8 min, they were exposed to a single BOP using an Advanced Blast Simulator (ABS^®^, ORA Inc., Fredericksburg, VA, USA). The ABS^®^ consists of a 0.5 ft long compression chamber that is separated from a 21 ft long transition/expansion test section by rupturable VALMEX^®^ membranes (Mehler Texnologies, Martinsville, VA, USA). Anesthetized mice were secured in a transverse side-on orientation in the test section and were exposed to a single 20 psi BOP using the high-fidelity ABS^®^ that mimics a “free-field”-like primary blast.

For immunosuppression, mice (*n* = 15) received dosages of 150 mg/kg and 100 mg/kg of cyclophosphamide (CP) per body weight through intraperitoneal (i.p.) injections on days minus 4 and minus 1 before wounding and infection, respectively.

For the surgical procedure, mice were anesthetized with intraperitoneal injections of ketamine at 130 mg/kg (Ketaset; Fort Dodge Animal Health, Fort Dodge, IA, USA) and xylazine at 10 mg/kg (AnaSed; Lloyd Inc., Shenandoah, IA, USA). Subcutaneous injections of SR-buprenorphine at 0.05 mg/kg (Covetrus North America. LLC, Elizabethtown, PA, USA) were used for pain management. Hair was clipped from the cervical to mid-lumbar dorsum, and the skin was scrubbed with an iodine solution followed by an isopropanol rinse. A 6.0 mm disposable skin biopsy punch (VisiPunch; Huot Instruments, LLC, Menomonee Falls, WI, USA) was used to create a full-thickness skin defect overlying the thoracic spinal column and the adjacent musculature. To simulate combat muscle trauma, three 2–3 mm^2^ areas of exposed paraspinal muscles were injured three times either by pinching with the sharp ends of surgical forceps or making deep cuts with a scalpel. For each animal group infected with *R. arrhizus* and *L. corymbifera* (*n* = 5 for the low-inoculum experiment and *n* = 4 for the high-inoculum experiment), aliquots of 25–100 μL of the respective fungal suspensions in sterile 0.9% saline were pipetted into the wound and allowed to absorb for 3 min. Control animals (*n* = 5 for the low-inoculum experiment and *n* = 4 for the high-inoculum experiment) received a similar volume of sterile 0.9% saline. A circular cutout (30 mm in diameter) of transparent dressing (TegadermRoll; 3M Health Care, St. Paul, MN, USA) was placed over the wound and secured with tissue adhesive (Vetbond; 3M Animal Care, St. Paul, MN, USA) for 5 days.

For treatment experiments, starting at 30 min post inoculation, mice (*n* = 6) were treated with L-AmB (30 mg/kg) (AmBisome^®^, Gilead Sciences, Inc., Foster City, CA, USA) for *R. arrhizus* hyphae-infected animals via IP injection once daily from day 1 to day 7 (8 treatments in total). Control animals from two separate experiments received either PBS (*n* = 8) or 5% dextrose solution (*n* = 6) via IP injection at the same time points. An additional control group (*n* = 8). received no infection 

### 2.6. Wound Assessment, Fungal Burden Semi-Quantification, and Histopathological Analysis

Wound images and wound surface area measurements were recorded on days 0, 3, 7, and 9 only for the initial experiments with low-hyphae-inoculum infections (with CP or BOP) and on days 0, 5, and 8 for all further experiments using the planimetry system Silhouette (Aranz Medical, Christchurch Central City, New Zealand). On day 8 or 9 post infection, all mice were humanely euthanized with CO_2_ anesthesia. Wound beds with muscle tissue were resected, split into two equal portions, and processed for the semi-quantification of fungal burden or histopathological analysis. ~10^7^ to 10^9^ CFUs of *R. arrhizus* or *L. corymbifera* hyphae were used for wound infection. For semi-quantification analysis, in the first experiment, the first portions of wound tissue (skin/muscle) from each animal were further divided into four pieces, which were plated with RPMI agar and overlayed with 1 mL of RPMI. In the second experiment, wound tissues for fungal cultures were either subjected to 10 s homogenization with hand-held TissueRuptor (Qiagen LLC, Germantown, MD, USA) or by gentle mincing with a scalpel, vortexing in 200 μL of RPMI medium for 5 s. We found that even brief tissue homogenization with a hand-held Qiagen device is detrimental for the majority of *R. arrhizus* skin/muscle samples (Figure S2). Similar data were reported by Dos Santos et al. (2019). They reported that homogenization of the brain, liver, lung, and spleen did not recover *R. arrhizus* colonies on Sabouraud Dextrose Agar (SDA) plates while culturing of ten fragments (2 × 2 mm) of the same organs resulted in the growth of viable fungi [34]. We found that our method combining gentle mincing with short vortexing is advantageous for semi-quantitative culturing. After sample processing, serial dilutions of suspensions were plated on RPMI agar and incubated at 30 °C overnight. Then, plates were incubated at room temperature and growth was monitored for up to 5 days. For histopathological analysis, the second portion of the collected wound tissues (skin/muscle) was fixed in 10% neutral buffered formalin, dehydrated, embedded in paraffin, and sectioned into 5 μm slices. The sections were stained with hematoxylin and eosin (H&E) or Grocott’s methenamine silver (GMS) and examined by light microscopy for tissue invasion, necrosis, and the presence of hyphae.

### 2.7. Statistical Analysis

Data are presented as means ± standard deviation of the mean. GraphPad Prism version 9.5.1.733 for Windows, GraphPad Software (www.graphpad.com) was used for statistical analysis, unpaired *t*-tests, two-way ANOVA, and Bonferroni’s adjustment for multiple comparisons tests as appropriate. A *p*-value of <0.05 was considered significant for all comparisons. All in vivo experiments were performed with at least five mice per group and were replicated at least two times.

## 3. Results

### 3.1. Hyphae of R. arrhizus and L. corymbifera Cause Sustained Wound Infection in Blast-Exposed and Cyclophosphamide-Treated Mice

**Figure 1:**
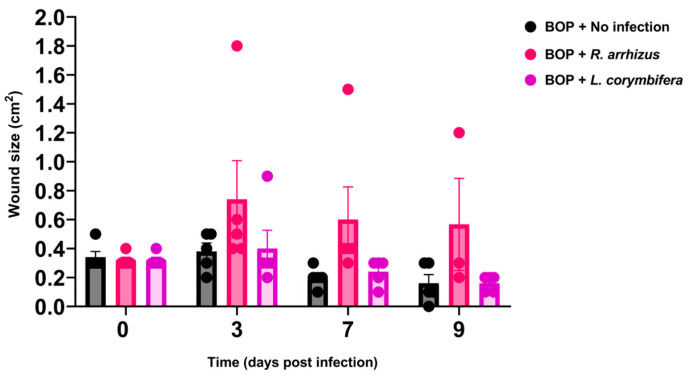
Effect of *Mucorales* infection induced in blast-exposed mice with low hyphal inocula of *Rhizopus arrhizus* or *Lichtheimia corymbifera* on wound size. Data are presented as mean ± SD. *p*-values were calculated using two-way ANOVA with Bonferroni’s multiple comparisons test and no significant differences were observed. Note: BOP: blast overpressure.

Our findings revealed that BOP exposure resulted in sustained wound infection (Figure 1) that was similar to the condition observed in CP-treated mice when challenged with 1.11 × 10^7^ *R. arrhizus* hyphae or 1.21 × 10^7^ *L. corymbifera* hyphae (Figure S3; Table 1). Due to CP-mediated immunosuppression, five animals in the no-infection control group developed an unknown infection and died within the first few days. Two mice from the BOP + *R. arrhizus* group developed severe ulcers outside the infection site and had to be euthanized before day 9. Additionally, a substantial enlargement of the wound in one *R. arrhizus*-infected animal was observed, compared to the other infected and non-infected animals. For the rest of the animals in the BOP + *R. arrhizus* group, there was a trend of larger wounds compared to the control no-infection group. All but one group (BOP + *R. arrhizus* − 67%*)* had a 100% infection rate (Table 1). However, our infection model was 100% non-lethal with no signs of distress. Histopathological analysis detected hyphae in fewer animals: only 33% of animals had visible hyphae in the infected wound tissues in both the BOP + *R. arrhizus* and BOP + *L. corymbifera* groups (Table 1). In the CP + *L. corymbifera* group, two of three animals were determined to have hyphae in the infected wound tissues.

For *R. arrhizus* infection, necrosis was evident in the panniculus carnosus muscle and subcutaneous tissues, accompanied by superficial pyogranulomatous dermatitis and fibrosis. Sparse fungal hyphae covered by a serocellular crust were observed amidst moderate necrotizing dermatitis (Figure S4). Similarly, infection with *L. corymbifera* was characterized by predominantly multifocal to coalescing pyogranulomatous and eosinophilic dermatitis and panniculitis. Degeneration of the panniculus carnosus muscle was evident, along with observations of multinucleated giant cells within the inflammatory areas, occasionally accompanied by fungal elements (Figure S4). Thus, infection induced a sustained wound mucormycosis that was characterized by necrosis of skin and muscle tissues, albeit with a lack of clinical scores and a statistically significant increase in wound size.

### 3.2. Higher Hyphal Inocula of R. arrhizus and L. corymbifera Result in Larger Wounds and More Extensive Tissue Necrosis in Blast-Exposed Mice

**Figure 2:**
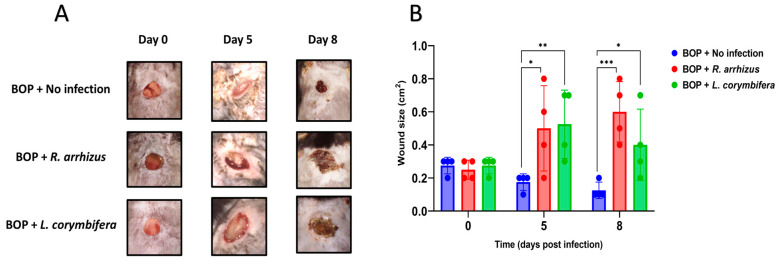
High inocula of *Rhizopus arrhizus* and *Lichtheimia corymbifera* hyphae cause severe wounds in a combat-relevant mouse model. (**A**). Images of wounds depict a progressive increase in wound size observed on days 0, 5, and 8. (**B**) Statistically significant increase in wound area observed on days 0, 5, and 8 in wounds infected with *R. arrhizus* or *L. corymbifera* hyphae compared to non-infected wounds. Data are presented as mean ± SD. *p*-values were calculated for using two-way ANOVA with Bonferroni’s multiple comparisons test (* *p* = 0.015, *** *p* < 0.001 for *R. arrhizus*; * *p* = 0.046, ** *p* = 0.008 for *L. corymbifera*). Note: BOP: blast overpressure.

**Figure 3:**
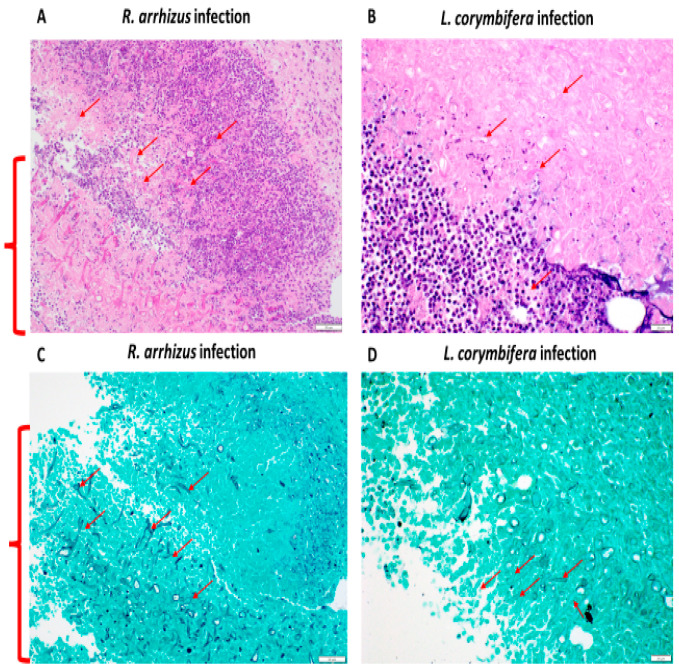
Histopathological analysis of wound bed infected with a high inocula of *Rhizopus arrhizus* and *Lichtheimia. corymbifera.* (**A**,**B**) Wound tissue stained with hematoxylin and eosin; (**C**,**D**) wound tissue stained with Grocott’s methenamine silver. Hyphae are shown with red arrows. Abundance of fungal elements is shown in red curly brackets. Scale: 50 µm.

We hypothesized that an increase in hyphal inoculum may result in more severe disease. Indeed, inoculating 5.40 × 10^8^ *R. arrhizus* and 2.65 × 10^8^ *L. corymbifera* hyphae resulted in more pronounced disease, leading to significantly larger wounds on day 5 and day 8 compared to non-infected wounds (Figure 2A,B). Visual inspection of the wounds revealed necrosis, severe skin inflammation, erythema, and swelling around the wound area. Most infected wounds presented abscesses during tissue collection. Plating of the wound tissues showed that all animals infected with *R. arrhizus* and 75% of those infected with *L. corymbifera* had viable fungal cells (Table 2). Notably, tissue homogenization led to very poor survival of *R. arrhizus* (25%) but not *L. corymbifera* (100%) (Figure S2). Consequently, we developed a semi-quantitative method to assess the quantification of *R. arrhizus* hyphal burden in hyphae-infected wound tissues that included a gentle mincing and vortexing the collected wound tissues. This revised method without homogenization achieved 100% hyphal viability (Figure S2). Histopathological analysis also showed 100% of *R. arrhizus*-infected animals had numerous hyphae within necrotic foci (Figure 3). Two out of four animals infected with *L. corymbifera* had hyphae within the necrotic dermis (Table 2).

Histopathological analysis of the H&E-stained tissues revealed more severe tissue damage compared to infections with lower inocula of *R. arrhizus* and *L. corymbifera* (Figure 3). Higher-inoculum *R. arrhizus* infections exhibited deeper extension of necrosis and inflammation beyond the panniculus carnosus muscle (Figure 3). Similarly, higher inoculum *L. corymbifera* infections displayed significant extension of inflammation and necrosis beyond the muscle layer, with aggregates of hyphae overlaying extensive necrotic areas (Figure 3).

### 3.3. Assessment of Liposomal Amphotericin B Efficacy against R. arrhizus Hyphae Infection in a Combat-Relevant Mouse Model

**Figure 4:**
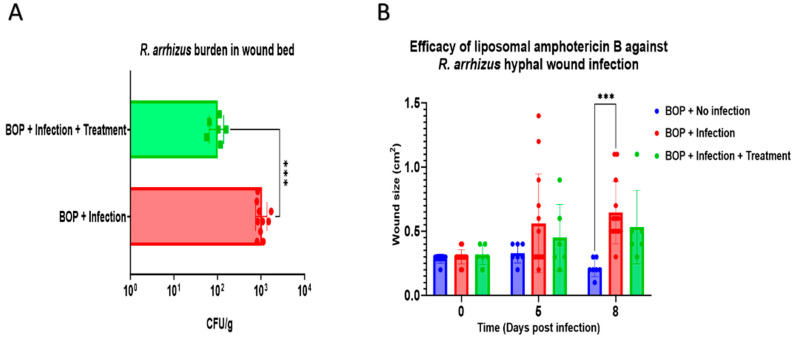
Efficacy of liposomal amphotericin B (L-AmB) against wound *Rhizopus arrhizus* infection. (**A**). Semi-quantification of wound tissues resulted in a significant decrease in colony-forming units in *R. arrhizus* hyphae infected wound tissue treated with L-AmB compared to infection alone. (**B**). No significant difference in wound size between infection alone and infection with treatment. Data are presented as mean ± SD. *p*-values were calculated for (**A**) using an unpaired *t*-test (*** *p* < 0.001) and for (**B**) using two-way ANOVA with Bonferroni’s multiple comparisons test (*** *p* < 0.001). Note: BOP: blast overpressure.

**Figure 5:**
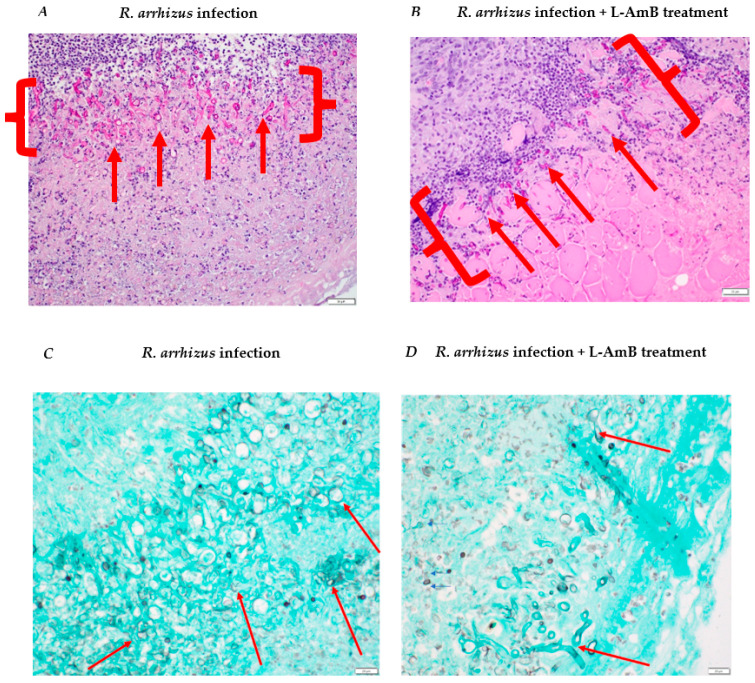
Histopathological analysis of effect of liposomal amphotericin B (L-AmB) treatment on wound mucormycosis caused by *Rhizopus arrhizus*. (**A**,**B**): Wound tissues stained with hematoxylin and eosin; scale—50 µm. (**C**,**D**): Wound tissues treated with Grocott’s methenamine silver staining; scale—20 µm. Region with red arrows indicating hyphal elements is present within necrotic areas in infected groups. Abundance of hyphae is indicated with red curly brackets.

We investigated the efficacy of L-AmB against severe *R. arrhizus* hyphae infection using our newly established combat-relevant wound infection mouse model. Wounds of blast-exposed mice were infected with a 5.03 × 10^9^ R. arrhizus hyphae inoculum and treated with L-AmB as described above. The semi-quantification of fungal burden in wound tissues revealed a significant reduction in the L-AmB-treated group compared to the infection-alone group (*p* < 0.001) (Figure 4A). As in the previous experiment, the vehicle-treated group showed a statistically significant difference in wound size (*p* < 0.001) compared to the non-infected group (Figure 4B). Although no significant difference in wound closure was observed between infected mice and those treated with L-AmB, a trend towards reduced wound size was noted in the treatment group. Notably, CFUs were detected in RPMI agar plates within 24 h of incubation in tissues from mice with infection alone, whereas L-AmB-treated mice displayed no detectable CFUs until 48 h, and less than six CFUs were detected within 72 h. These observations were corroborated by the level of tissue infection (Table 3), in which wounds infected with *R. arrhizus* post-BOP exposure revealed extensive necrosis across the dermis, subcutis, panniculus carnosus muscle, and deeper tissues (Figure 5A). Hyphal growth was notably widespread within these necrotic areas, infiltrating normal and degenerating myocytes (4/6 mice) and indicating substantial tissue invasion and damage (Figure 5A,C). Following L-AmB administration post infection, necrosis primarily affected the subcutis and underlying tissues (Figure 5B). Notably, the presence of hyphae was confined mainly to necrotic regions with no growth over myocytes (6/6 mice), suggesting a potential impact of the treatment on constraining fungal spread beyond these specific areas (Figure 5B,D). 

## 4. Discussion

We report on the development of a murine model of wound mucormycosis with hyphae inoculation. This model recapitulates penetrating wounds with skin, fascia, and muscle tissue injuries following traumatic fungal inoculation. The addition of a blast overpressure (BOP) exposure mimics battlefield or combat conditions.

Post-traumatic direct/environmental inoculation of molds into wounds is hypothesized to be the leading cause of WIFDs in military and civilian patients as fungal colonies are often found in hyphal biofilms and mycelia in soil [15]. There are several lines of circumstantial evidence that suggest *Mucorales* hyphae are involved in fungal wound infections. First, *Saksenaea* spp. and *Apophysomyces* spp., which caused two-thirds of all angio-IFDs in U.S. military casualties in Afghanistan [36], are very poor sporangiospore producers under different experimental conditions [37]. Second, a very high inoculum of *R. arrhizus* sporangiospores (5 × 10^7^) was reported/required for the development of infection in severely immunocompromised mice [28]. It is unlikely that such a heavy conidial contamination of wounds of human patients could come from airborne sporangiospores. Lastly, we have recently shown that hyphae of *R. arrhizus* and *L. corymbifera* are 10 and 16 times more lethal in a *G. mellonella* infection model compared to sporangiospores [33]. Thus, due to a strong hypothesis of wound infection development with hyphae, these fungal elements were used in our study.

In nature, saprobic filamentous fungi, including *Mucorales,* form mycelial structures that represent an important source of organic matter in different types of soil [38,39,40,41]. A recent video depicts a *Mucor* mycelium growing in soil [42]. Formation of biofilms composed of hyphae of *Mucorales*, and other saprobic filamentous fungi is also well documented [43,44,45,46,47]. Soil *Mucorales* mycelia/biofilms can readily infect wounds. For example, a 45-year-old man who rolled on the ground to extinguish a fire developed burn wound mucormycosis caused by *Apophysomyces elegans*. Multiple soil samples from the ground of the accident yielded colonies of *A. elegans* that were morphologically very similar to the patient’s isolate [48]. Strikingly, an aggregate of *Mucor hiemalis* hyphae that may represent soil mycelia or biofilm was found in the dermis of a patient with a traumatic wound acquired during gardening [49]. When combat wounds sustained from penetrating blast injuries are contaminated with foreign bodies, such as soil, plants, wood, gravel, and glass, we hypothesize that the organism is inoculated predominantly in the form of mycelia and hyphal biofilm. While sporangiospores may also constitute part of the soil mycelia, hyphae comprise the bulk of this biomass [38]. Therefore, we speculate that although sporangiospores of *Mucorales* and other pathogenic invasive molds may be present within a mycelium, they would likely constitute a small proportion of the blast-injected fungal inoculum.

The traumatic compromise of the skin and fascia is one of the major factors in the initiation of bacterial and fungal wound infections; however, the host immune system controls the development of sustained infection of opportunistic pathogens. Exposure of male BALB/c mice to BOP was recently reported to result in immune system dysfunction [29]. Our model produced a sustained fungal infection for up to 9 days in 67–100% of C57BL/6 mice when infected with approximately 10^7^ of germinated sporangiospores/hyphae. However, histopathological analysis revealed that only a few fungal elements were located superficially within necrotic tissues. Infections with 20–50-fold higher inocula of *L. corymbifera* or *R. arrhizus* resulted in large wound size, extension of necrosis, and abundant identification of hyphae throughout the skin/muscle tissues. The clinical presentation was consistent with previously reported pyogranulomatous-to-necroulcerative dermatitis and panniculitis [50]. However, even with such high inocula leading to an increased presence of hyphal elements, there was no deep penetration of tissues or invasion of large vessels, which are typically characteristic of severe wound mucormycosis and seen in casualties with extensive trauma. While wound infection is likely to be contained when the trauma is minor and there are no underlying diseases, major trauma, including blast exposure, that leads to recurring sepsis, shock, and multiple blood transfusions creates an immune-deficient state or immune paralysis, predisposing individuals to WIFDs [13,15,16,31]. In post-traumatic wounds, hyphae of *Mucorales* may extend into the deep fascia and muscle layers, infiltrating blood vessels and leading to tissue hypoxia and subsequent necrotizing fasciitis with a characteristic black eschar [15,51,52]. We hypothesize that the modest changes in immune response caused by the experimental BOP in our study are not comparable to the dramatic immune system dysregulation associated with severe trauma caused by blast munitions on the battlefield. In Afghanistan, soldiers with WIFDs who had high index severity scores (greater than 21) often underwent amputations and multiple blood transfusions, which would all result in severe immune system dysregulation [9,53,54]. During the Serbian conflict, blast casualties showed profound changes in both pro-inflammatory and anti-inflammatory cytokines [6], as compared to modest changes in cytokine/chemokine levels in mice subjected to 20 psi BOP [29]. Additionally, in our mouse model, wounds were not as large and deep as in combat casualties. WIFDs in Service Members were reported to be associated with limb amputations [9]. Nonetheless, the tissue injury, wound necrosis, hyphal invasion, and quantitative hyphal viability in the setting of BOP injury is a model system that approximates mucormycotic combat WIFD as a pre-clinical platform for evaluating new antifungal or immune augmenting therapeutics.

Our study also marks the first investigation evaluating first-line antifungal L-AmB against a murine combat-relevant wound mucormycosis. L-AmB treatment resulted in wound size reduction, though this difference was not statistically significant. Notably, this treatment substantially reduced *R. arrhizus* burden in wound bed tissue, evidenced by CFU counts. This difference indicates a fungicidal effect of L-AmB, either alone or in combination with the immune response. However, L-AmB was not effective in completely eliminating *R. arrhizus* wound infection. For the treatment group, histological analysis identified that hyphal elements were present in the necrotic skin tissue but were absent in underlying muscle tissue. The disruption of blood supply to the necrotic tissues may limit therapeutic effects. In a single previous report on the treatment of cutaneous mucormycosis in an animal model [28], oral treatment with 40 mg/kg posaconazole (POS), a standard of care azole antifungal, resulted in a significant reduction in lesion size caused by *R. arrhizus* infection in mice. However, POS treatment was not effective in fungal load reduction unless used in combination with tacrolimus. Even after combination therapy, a considerable fungal load was detected in the tissues of the treated animals. Perhaps, similar to our study, fungi survived in the necrotic tissues. The addition of topical treatment may contribute to a further reduction in viable fungi and to enhanced wound closure.

We acknowledge the limitations of our study, including our reliance on a single isolate of *R. arrhizus*. During the model’s development, *L. corymbifera* inconsistently produced a sustained wound infection compared to *R. arrhizus*. Notably, it was previously reported that *R. arrhizus* was maintained for at least 7 days, while *M. irregularis* was detected for only 3 days in the skin of footpads of immunocompetent C57BL/6 mice [20,21]. Moreover, we used a hyphal inoculum prepared in a laboratory medium rather than a natural hyphal biofilm where the mycelium and sporangiospores can be present. Extracellular matrixes from biofilms may exhibit additional protection against an effective immune response. Also, foreign bodies, such as soil, plants, wood, rocks, and glass can be present in combat penetrating wounds that are sustained from blast explosion. Further investigation of the impact of fungal biofilm, extracellular matrices, and innate material on the pathogenesis and treatment of wound mucormycosis is warranted. Moreover, the degree of simulated immune impairment may be enhanced to more closely mimic the profound immune dysregulation of military-related blast injuries and penetrating trauma. The impact of radiation injury and burns are other forms of injury that warrant further investigation. Additionally, only one antifungal agent was used as a systemic monotherapy. Future studies should address combinations of licensed antifungal agents, investigational drugs, immune augmentation, and topical treatments. The role of hyperbaric oxygen also warrants investigation in combination with optimizing antifungal therapeutics.

In summary, to our knowledge, we developed the first murine model for combat-relevant invasive mucormycosis, providing a valuable tool for evaluating the efficacy of potential therapeutic interventions against the threat of fungal infections to wounded military personnel. Additionally, this work demonstrated the successful use of hyphae of two species, *R. arrhizus* and *L. corymbifera*, to induce sustained infections in a combat-relevant animal model. This model was further validated by demonstrating L-AmB’s efficacy against wound mucormycosis. While further research is needed to elucidate combination therapeutics and explore alternative treatment options, this study lays a foundation for developing effective strategies to combat WIFDs in the context of military operations.

## Figures and Tables

**Table 1:** Effect of low inocula of *R. arrhizus and L. corymbifera* on development of infection in blast-exposed or cyclophosphamide-treated mice.

Mouse Groups	Proportion of Infected Mice Detected by		Histological Analysis of Wound Tissues
	CFUs	Histology	
BOP + *R. arrhizus*	2/3	1/3	Necrosis of the panniculus carnosus muscle and subcutaneous tissues Superficial pyogranulomatous dermatitis and fibrosis Presence of rare hyphae Moderate necrotizing dermatitis Overlying serocellular crust
CP + *R. arrhizus*	5/5	1/3	Necrosis of the epidermis and dermis Presence of an overlying serocellular crust Inflammation extended to the underlying subcutis Inflammation reached the skeletal muscle
BOP + *L. corymbifera*	5/5	2/3	Multifocal to coalescing pyogranulomatous and eosinophilic dermatitis and panniculitis Degeneration of the panniculus carnosus muscle Multifocal pyogranulomatous and eosinophilic superficial dermatitis Presence of multinucleated giant cells Rare visibility of fungal elements
CP + *L. corymbifera*	4/4	1/3	Multifocal necrotizing dermatitis Panniculitis Myositis Absence of extension into underlying epaxial musculature

Note: CFUs: colony-forming units; BOP: blast overpressure; CP: cyclophosphamide.

**Table 2:** Effect of high inocula of *R. arrhizus* and *L. corymbifera* on wound infection in blast-exposed mice.

Organism	Proportion of Mice with Detectable CFUs/Tissues Infected			Histological Analysis of Wound Tissues
	With Homogenization	Without Homogenization	Histology	
*R. arrhizus*	1/4	4/4	4/4	Presence of numerous hyphae Admixture with necrotic material Extended into deep dermis
*L. corymbifera*	3/4	3/4	2/4	Hyphal aggregates in the deep dermis Abundant necrotic material overlying the aggregates

Note: CFUs: colony-forming units.

**Table 3:** Effect of *R. arrhizus* hyphae infection and liposomal amphotericin B treatment on tissue involvement and infection levels in blast-exposed mouse wounds.

Mouse Groups	Proportion of Mice with Detectable CFUs/Tissues Infected		Histological Analysis of Wound Tissues
	CFUs	Histology	
BOP + Infection	10/10	6/6	Necrosis in the dermis, subcutis, panniculus carnosus muscle, and underlying tissues Growth of hyphae in areas of necrosis Hyphae were spanning across normal and degenerating myocytes
BOP + Infection + Treatment	6/6	6/6	Necrosis of the subcutis and underlying tissues Hyphae were primarily located in areas of necrosis

Note: CFUs: colony-forming units; BOP: blast overpressure.

## Data Availability

Data are available upon request from the corresponding author.

## Supplementary Materials

The following supporting information can be downloaded at: https://www.mdpi.com/article/10.3390/jof10050364/s1. Figure S1: Comparison of two media for sporangiospore germination; Figure S2: Homogenization of wound tissues results in lost viability of *R. arrhizus* but not of *L. corymbifera*; Figure S3: Comparison of wound size in blast over pressure (BOP) exposed and cyclophosphamide (CP) treated mice that were infected with low inocula of *Rhizopus arrhizus* and *Lichtheimia corymbifera*. Figure S4: Histopathological analysis of wound beds infected with a low inocula of *Rhizopus arrhizus* and *Lichtheimia corymbifera* infections after blast overpressure exposure. Table S1: Group and number of animals subjected to each experimental condition.

## Disclaimers

This material has been reviewed by the Walter Reed Army Institute of Research. There is no objection to its presentation and/or publication. The opinions or assertions contained herein are the private views of the author, and are not to be construed as official, or as reflecting true views of the Department of the Army or the Department of Defense. Research was conducted under an IACUC-approved animal use protocol in an AAALAC International—accredited facility with a Public Health Services Animal Welfare Assurance and in compliance with the Animal Welfare Act and other federal statutes and regulations relating to laboratory animals.
