# Supplementary material for: Development of a Combat-Relevant Murine Model of Wound Mucormycosis: A Platform for the Pre-Clinical Investigation of Novel Therapeutics for Wound-Invasive Fungal Diseases
Source: J Fungi (Basel). 2024 May 20;10(5):364. doi: 10.3390/jof10050364 (PMC11122444; doi:10.3390/jof10050364)
Supplement: Supplementary file 1 [file jof-10-00364-s001.zip › jof-2968161-supplementary.pdf]

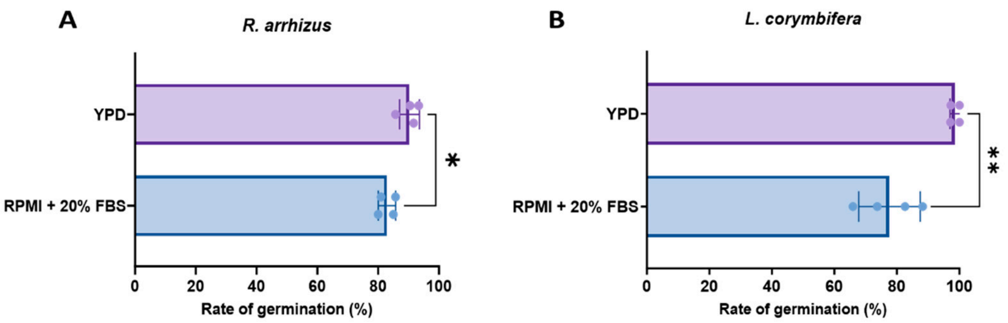

Figure S1. Comparison of two media for sporangiospore germination. Yeast Peptone Dextrose (YPD) medium significantly enhances the sporangiospore germination rate of *R. arrhizus* (* $p = 0.015$) (**A**) and *L. corymbifera* (** $p = 0.006$) (**B**) compared to RPMI + 20% FBS. The data are presented as Mean ± SD. *p*-value was calculated using an unpaired *t*-test.

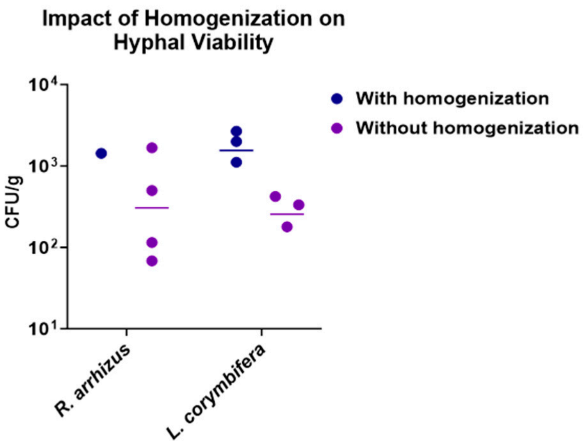

Figure S2. Homogenization of wound tissues results in lost viability of *R. arrhizus* but not of *L. corymbifera.*

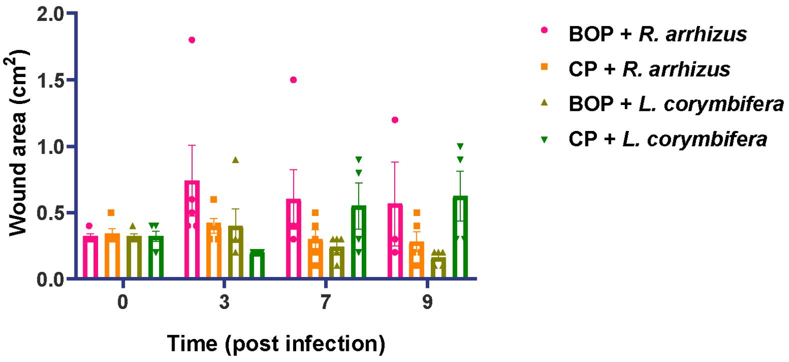

Figure S3. Comparison of wound size in blast over pressure (BOP) exposed and cyclophosphamide (CP) treated mice that were infected with low inocula of *Rhizopus arrhizus* and *Lichtheimia corymbifera.* The data are presented as Mean ± SD. *p*-values were calculated using two-way ANOVA with Bonferroni's multiple comparisons test and no significant differences were observed. Note: BOP: Blast Over Pressure; CP: Cyclophosphamide.

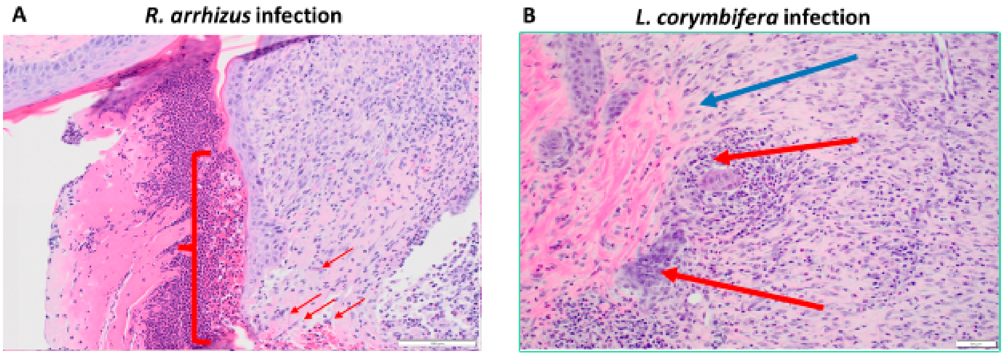

Figure S4. Histopathological analysis of wound beds infected with a low inocula of *Rhizopus arrhizus* and *Lichtheimia corymbifera* infections after Blast Overpressure exposure. (**A**,**B**) Cutaneous wound site stained with hematoxylin and eosin. (**A**) A serocellular crust overlies a region of epidermal ulceration and necrosis (red bracket). Subjacent to the ulceration, there is moderate pyogranulomatous and eosinophilic necrotizing dermatitis, with fibrosis and infrequent *R. arrhizus* hyphae (red arrows). (**B**)There is a distinct border between mature, pre-existing dermal collagen, and the younger collagen of granulation tissue (blue arrow). There is abundant pyogranulomatous and eosinophilic dermatitis, often oriented at the dermal-epidermal junction (red arrow), with leukocytes encircling rete pegs of epidermis and overlying granulation tissue. Scale: 50 µm.

Table S1. Group and number of animals subjected to each experimental condition.

**Experiment 1**			**10^7 hyphae**	
	**Group**	**No infection**	***R. arrhizus***	***L. corymbifera***
	BOP	$n = 5$	$n = 5$	$n = 5$
	CP	$n = 5$	$n = 5$	$n = 5$
**Experiment 2**			**10^8 hyphae**	
	**Group**	**No infection**	***R. arrhizus***	***L. corymbifera***
	BOP	$n = 4$	$n = 4$	$n = 4$
**Experiment 3 and 4**			**10^9 hyphae**	
	**Group**	**No infection**	***R. arrhizus***	**BOP + *R. arrhizus* + L-AmB (30 mg/kg)**
	BOP	$n = 8$	$n = 8 + 6$ *	$n = 6$

Note: BOP: Blast over pressure; CP: Cyclophosphamide; L-AmB: liposomal amphotericin B; *animals are from 2 separate experiments.
